# Longitudinal Associations of Mental Disorders With Dementia: 30-Year Analysis of 1.7 Million New Zealand Citizens

**DOI:** 10.1001/jamapsychiatry.2021.4377

**Published:** 2022-02-16

**Authors:** Leah S. Richmond-Rakerd, Stephanie D’Souza, Barry J. Milne, Avshalom Caspi, Terrie E. Moffitt

**Affiliations:** 1 Department of Psychology, University of Michigan, Ann Arbor; 2 Centre of Methods and Policy Application in the Social Sciences (COMPASS), University of Auckland, Auckland, New Zealand; 3 School of Social Sciences, University of Auckland, Auckland, New Zealand; 4 Department of Psychology and Neuroscience, Duke University, Durham, North Carolina; 5 Department of Psychiatry and Behavioral Sciences, Duke University School of Medicine, Durham, North Carolina; 6 Center for Genomic and Computational Biology, Duke University, Durham, North Carolina; 7 Institute of Psychiatry, Psychology, and Neuroscience, King’s College London, London, England; 8 Promenta Center, University of Oslo, Oslo, Norway

## Abstract

**Importance:**

Mental disorders are an underappreciated category of modifiable risk factors for dementia. Developing an evidence base about the link between mental disorders and dementia risk requires studies that use large, representative samples, consider the full range of psychiatric conditions, ascertain mental disorders from early life, use long follow-ups, and distinguish between Alzheimer disease and related dementias.

**Objective:**

To test whether mental disorders antedate dementia across 3 decades of observation.

**Design, Setting, and Participants:**

This population-based administrative register study of mental disorders and Alzheimer disease and related dementias included all individuals born in New Zealand between 1928 and 1967 who resided in the country for any time during the 30-year observation period between July 1988 and June 2018. Data were from the New Zealand Integrated Data Infrastructure, a collection of whole-of-population administrative data sources linked at the individual level. Data were analyzed from October 2020 to November 2021.

**Exposures:**

Diagnoses of mental disorders were ascertained from public-hospital records.

**Main Outcomes and Measures:**

Diagnoses of dementia were ascertained from public-hospital records, mortality records, and pharmaceutical records.

**Results:**

Of 1 711 386 included individuals, 866 301 (50.6%) were male, and individuals were aged 21 to 60 years at baseline. Relative to individuals without a mental disorder, those with a mental disorder were at increased risk of developing subsequent dementia (relative risk [RR], 4.24; 95% CI, 4.07-4.42; hazard ratio, 6.49; 95% CI, 6.25-6.73). Among individuals with dementia, those with a mental disorder developed dementia a mean of 5.60 years (95% CI, 5.31-5.90) earlier than those without a mental disorder. Associations held across sex and age and after accounting for preexisting chronic physical diseases and socioeconomic deprivation. Associations were present across different types of mental disorders and self-harm behavior (RRs ranged from 2.93 [95% CI, 2.66-3.21] for neurotic disorders to 6.20 [95% CI, 5.67-6.78] for psychotic disorders), and were evident for Alzheimer disease (RR, 2.76; 95% CI, 2.45-3.11) and all other dementias (RR, 5.85; 95% CI, 5.58-6.13).

**Conclusions and Relevance:**

In this study, mental disorders were associated with the onset of dementia in the population. Ameliorating mental disorders in early life might also ameliorate neurodegenerative conditions and extend quality of life in old age.

## Key Points

**Question:**

Do mental disorders antedate dementia in the population?

**Findings:**

In this population-based administrative register study of 1.7 million New Zealand citizens observed for 3 decades, people with early-life mental disorders were at elevated risk of subsequent dementia and younger dementia onset. Associations were evident across different psychiatric conditions, for Alzheimer disease and all other dementias, and after accounting for preexisting physical diseases and socioeconomic deprivation.

**Meaning:**

Findings from this study suggest that ameliorating mental disorders in early life might reduce risk of cognitive decline and neurodegenerative disease in later life.

## Teaser

This population-based administrative register study investigates whether mental disorders antedate dementia among 1.7 million New Zealand citizens across 3 decades of observation.

## Introduction

Old age brings risk for many physical diseases, but neurodegenerative conditions, including Alzheimer disease and related dementias, have a disproportionate impact on disability and loss of independence in older adults.^1^ Recognition of the outsized influence of dementia on later-life functioning has fueled research into modifiable risk factors and prevention targets.^2,3,4,5,6^

Mental disorders might comprise an underappreciated category of modifiable risk factors.^7^ A recent commission of dementia experts identified depression among the 12 preventable risk factors most robustly associated with dementia.^3^ Systematic reviews and meta-analyses have implicated other psychiatric conditions in dementia risk, including anxiety,^8,9^ schizophrenia,^10^ and bipolar disorder.^11^ However, most dementia risk indexes do not include mental disorders or count only depression.^3^

Prior research has provided important information about dementia’s psychiatric antecedents. However, with few exceptions,^12,13^ most prospective studies have observed individuals from midlife or later, limiting the ability to capture mental disorders during their earlier life period of peak prevalence and compare associations with early-onset vs later-onset dementia. Further, most research has focused on 1 mental disorder at a time. The few studies to evaluate more than 1 disorder have grouped dementia outcomes with other organic disorders,^13^ used retrospective case-control designs,^14^ or observed individuals from midlife.^15^ Evaluating the full range of psychiatric conditions prospectively and from early life and ascertaining dementia outcomes specifically is necessary to differentiate generalized from disorder-specific risk factors over the life span. Here, we address these gaps by leveraging nationwide health register data on 1.7 million New Zealand citizens aged 21 to 60 years at baseline to track associations between the complete range of mental disorders and subsequent dementia across 3 decades.

An association between mental disorders and dementia would provide a basis for basic science and epidemiologic studies aiming to clarify its underlying mechanisms. The association could result from mechanisms precipitated by psychiatric problems, such as poor health behaviors, social isolation, effects of psychiatric medications, or downward socioeconomic drift.^16,17,18,19^ An association might also arise from shared causes, including genetic risk, low childhood IQ, low education, neuroinflammation, oxidative stress, or cerebral small-vessel disease.^3,20,21,22,23,24,25^ Even if the association is not causal, establishing a prospective link would identify psychiatric conditions as an early warning sign for neurodegenerative disease.

We tested the hypothesis that mental disorders are associated with dementia in the population. We tested whether associations are present across different psychiatric conditions and for Alzheimer disease and all other dementias. We also tested whether associations held after controlling for preexisting chronic physical diseases.

## Methods

Data were from the New Zealand Integrated Data Infrastructure, a collection of deidentified, individually linked, whole-of-population administrative data sources.^26,27^ Ethical approval was obtained from the University of Auckland Human Participants Ethics Committee. Output data underwent confidentiality review by Statistics New Zealand Tatauranga Aotearoa. Informed consent was not obtained per rule 11(2)(c)(iii) of the New Zealand Health Information Privacy Code,^28^ which, under certain circumstances, allows for anonymized health data to be used for research without the authorization of the individual concerned. This study followed the Strengthening the Reporting of Observational Studies in Epidemiology (STROBE) reporting guideline.

### Study Population

Our study population included the 1 711 386 individuals aged 21 to 90 years who were born in New Zealand between 1928 and 1967 and who resided in New Zealand for any time between the July 1988 and June 2018 fiscal years. We selected this age range to capture as much of the period of peak prevalence for mental disorders as possible, while also capturing the period of risk for dementia (including early-onset conditions) during the 30-year period. We excluded individuals who were not born in New Zealand; were born in New Zealand outside of 1928 to 1967; or were born in New Zealand between 1928 and 1967 but died before the observation period or were overseas during the entire period. We divided the population into age-bands (born from 1928-1937, 1938-1947, 1948-1957, and 1958-1967).

### Mental Disorders

We collected information about mental health admissions to public hospitals from records maintained by the New Zealand Ministry of Health. Public hospitals administer most of New Zealand’s medical treatment.^29^ They provide acute and nonacute services (including medical, surgical, maternity, diagnostic, and emergency services) for mental and physical health concerns. We collected information about primary diagnoses, external cause codes, and procedure codes for admissions based on *International Statistical Classification of Diseases and Related Health Problems, Tenth Revision *(*ICD*-*10*) and corresponding diagnoses in *ICD-9*. We classified 9 broad categories of mental disorders: substance use, psychotic, mood, neurotic (ie, anxiety), physiological disturbance, personality, developmental, behavioral, and unspecified disorders. We also obtained data about self-harm. Details of diagnoses and their coding are reported previously^30^ and in eAppendix 1 in the Supplement.

### Alzheimer Disease and Related Dementias

We collected information about Alzheimer disease and related dementia diagnoses using a previously published scheme.^31^ Dementias were ascertained via *ICD*-*10* and corresponding *ICD*-*9* dementia codes in public hospital and mortality records maintained by the New Zealand Ministry of Health and antidementia drug prescriptions in pharmaceutical records maintained by the New Zealand Pharmaceutical Management Agency (eAppendix 2 in the Supplement). Hospital records were available for the 30-year observation period (July 1988 to June 2018), mortality records were available from July 1988 to December 2016, and pharmaceutical records were available from July 2006 to June 2018. Although dementia in the community was likely underidentified in our medical register–based ascertainment scheme, cases were classified accurately: 83.1% of cases diagnosed with dementia in medical registers were also diagnosed in community-based assessments (eAppendix 3 in the Supplement).

### Physical Diseases

We obtained information about 8 physical diseases classified as chronic by the New Zealand Ministry of Health, including coronary heart disease, gout, chronic obstructive pulmonary disease, diabetes, cancer, traumatic brain injury, stroke, and myocardial infarction. Details of diagnoses and their coding are reported previously^30^ and in eAppendix 4 in the Supplement.

### Statistical Analysis

We used Poisson regression models with relative risks (RRs) to estimate the associations between a diagnosis of any mental disorder, a diagnosis of any physical disease, and subsequent dementia. We estimated bivariate associations and multivariate associations in which mental disorders and physical diseases were entered together as predictors.

To conduct a fine-grained analysis of the temporal association between mental disorders and dementia, we used Poisson regression models with RRs and Cox proportional hazards models (with censoring for out-migration or death from causes other than dementia) to estimate the associations between individuals’ index mental disorder (first diagnosed mental disorder during the observation period) and subsequent dementia, controlling for physical diseases diagnosed before the index disorder. We used generalized linear models to obtain unadjusted and covariate-adjusted estimates (least-squares means) of mean time to dementia diagnosis among individuals with and without a mental disorder diagnosis. In the Cox and generalized linear models, time since the index mental disorder (in days) was the time variable.

We analyzed associations for all mental disorders together and separately for each disorder type. We analyzed associations for all dementias together and separately for Alzheimer disease vs other dementias. To account for differing durations of observation time between mental disorder cases (observed from their first mental health hospitalization) and controls (all observed for 30 years with no mental disorder), we randomly assigned observation periods to controls to match observation durations among cases (eAppendix 5 in the Supplement).^30^ To preclude reverse causation, analyses excluded the 5559 individuals who had dementia prior to their mental health diagnosis or prior to the start of their matched observation period. We weighted data for Poisson regression analyses based on time alive and in New Zealand to account for remaining differences between individuals in observation time owing to death or out-migration. We conducted sensitivity analyses to test whether associations were robust to exclusion of pharmaceutical and mortality records from the dementia ascertainment scheme, use of unweighted data, and control for neighborhood deprivation as a proxy for social class.

Associations were estimated within the total population and by age band and sex. Models using the total population controlled for sex and birth year. Per the confidentiality rules of Statistics New Zealand, reported counts were randomly rounded to a base of 3. Therefore, counts do not always sum to totals. Statistical analysis was performed using SAS Enterprise Guide version 7.1 (SAS Institute).

## Results

### Distributions of Mental Disorders and Dementia

The study population included the 1 711 386 individuals (866 301 [50.6%] men and 845 085 [49.4%] women; aged 21 to 60 years at baseline) who were born in New Zealand between 1928 and 1967 and resided in New Zealand for any period between July 1988 and June 2018. Of these individuals, 252 981 (14.8%) were born between 1928 and 1937, 356 232 (20.8%) between 1938 and 1947, 500 673 (29.3%) between 1948 and 1957, and 601 494 (35.1%) between 1958 and 1967.

During the 30-year period, 64 857 individuals (3.8%) were identified as having a mental disorder and 34 029 (2.0%) were identified as having dementia. Similar percentages of men and women and more younger than older individuals were identified as having a mental disorder. Similar percentages of men and women and more older than younger individuals were identified as having dementia (eAppendix 6 in the Supplement).

**Figure 1.**
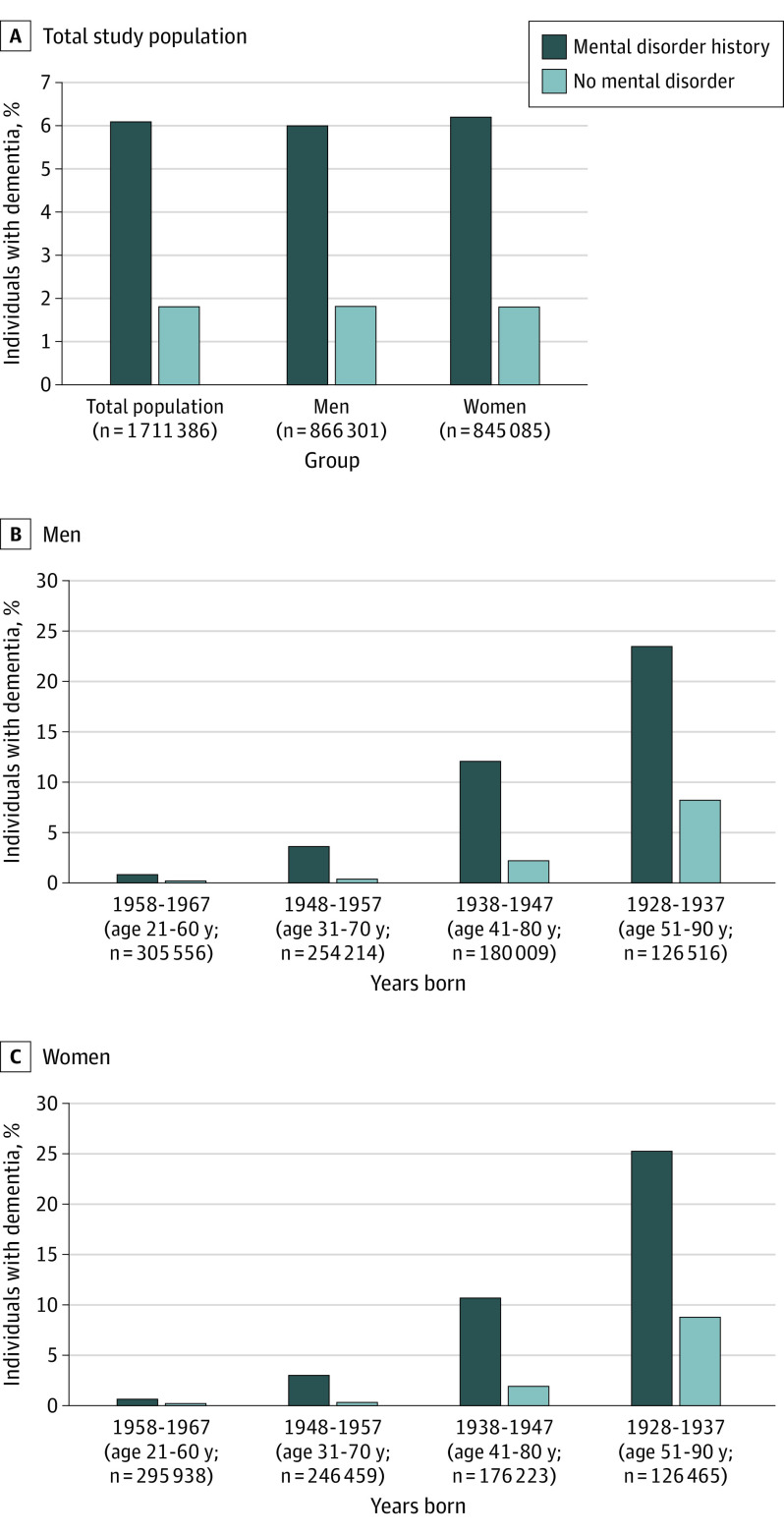
Overrepresentation of Dementia Among Individuals With a Mental Disorder The prevalence of dementia diagnoses was higher among individuals diagnosed with a mental disorder than among those without a mental disorder diagnosis. This was the case in the total population (A) and among men (B) and women (C) of all ages. Prevalence estimates were calculated over the 30-year observation period. Counts were randomly rounded to a base of 3 per the confidentiality rules of Statistics New Zealand. Therefore, counts do not always sum to totals. Age ranges indicate ages during the 30-year observation period.

Dementia was overrepresented among individuals with a mental disorder: of individuals diagnosed with a mental disorder, 6.1% (3957 of 64 857) were also diagnosed with dementia during the observation period, compared with 1.8% of individuals (30 072 of 1 646 529) without a mental disorder (Figure 1A). Dementia was overrepresented among mental disorder cases in men, women, and all age bands (Figure 1B and C).

### Associations of Mental Disorders With Subsequent Dementia

**Figure 2.**
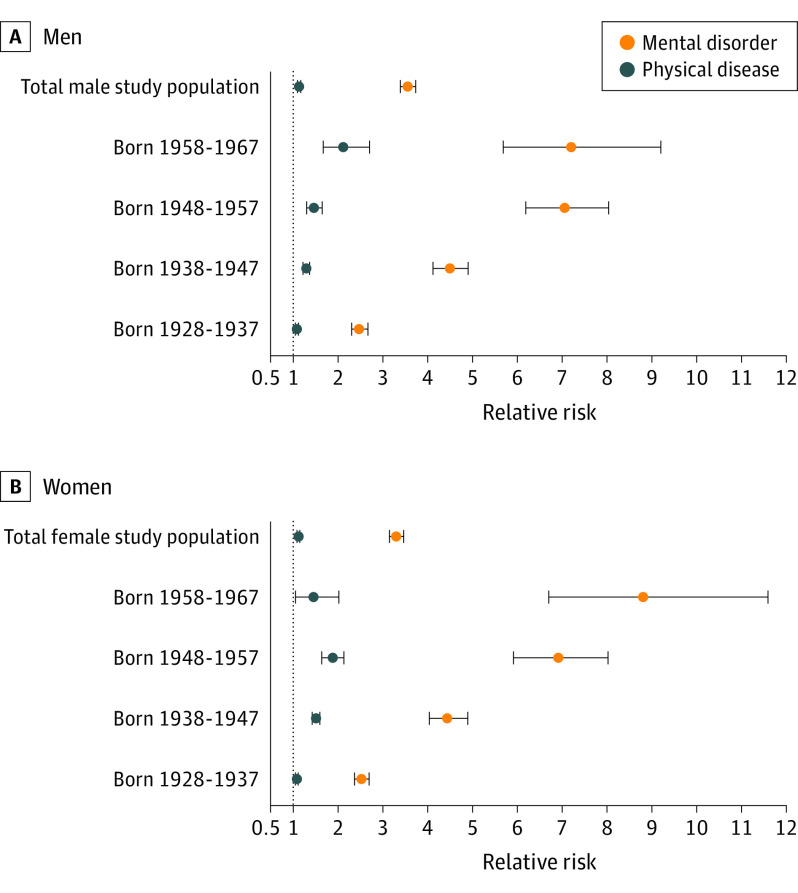
Associations Between Mental Disorder Diagnoses and Subsequent Dementia and Chronic Physical Disease Diagnoses and Subsequent Dementia Both mental disorders and chronic physical diseases were associated with subsequent dementia, but associations for mental disorders were larger than those for physical diseases. This was the case among men (A) and women (B) of all ages. Estimates are from multivariate models in which mental disorders and physical diseases were entered together as predictors. We ascertained mental disorder and physical disease diagnoses during the 30-year observation period. To be included in analyses, dementia diagnoses had to occur subsequent to the mental disorder or physical disease diagnosis. Models in which data were combined across cohorts (within sex) controlled for birth year. Error bars indicate 95% CIs.

Individuals diagnosed with a mental disorder were more likely to develop dementia than those without a mental disorder diagnosis (RR, 3.51; 95% CI, 3.39-3.64). The association between mental disorders and dementia was larger than the association between physical diseases and dementia (RR, 1.19; 95% CI, 1.16-1.21). Associations with mental disorders were evident in men, women, and all age bands (Figure 2; eAppendix 7 in the Supplement). Associations were stronger among more recently born cohorts (Figure 2; eAppendix 7 in the Supplement).

The association between mental disorders and dementia was equally evident when we observed individuals from their index mental disorder and equated mental disorder cases and controls on observation time. Even after accounting for preexisting physical illnesses, those with a mental disorder remained at elevated risk of developing dementia (RR, 4.24; 95% CI, 4.07-4.42; HR, 6.49; 95% CI, 6.25-6.73), and increased risk was evident across different lengths of follow-up from the index mental disorder (eAppendix 8 in the Supplement). Among individuals diagnosed with dementia, those diagnosed with a mental disorder developed dementia a mean of 5.60 (95% CI, 5.31-5.90) years earlier than those without a mental disorder diagnosis (mean time to dementia: with a mental disorder, 8.56 years; 95% CI, 8.29-8.84; with no mental disorder, 14.17 years; 95% CI, 14.07-14.27; eAppendix 9 in the Supplement). This association was observed across sex and age (Table). Associations were stronger among more recently born cohorts (Table).

**Table.** Associations Between Mental Disorders and Subsequent Dementia in the New Zealand Population^a^

Birth year; age range, y	Total, No.	Men			Women		
		No. (%)	Relative risk (95% CI)	Hazard ratio (95% CI)	No. (%)	Relative risk (95% CI)	Hazard ratio (95% CI)
Born 1958-1967; aged 21-60 y	601 404	305 502 (50.8)	10.25 (7.84-13.39)	11.66 (9.03-15.05)	295 902 (49.2)	10.83 (7.98-14.69)	12.14 (9.06-16.28)
Born 1948-1957; aged 31-70 y	500 349	254 034 (50.8)	9.36 (8.11-10.79)	11.25 (9.80-12.91)	246 315 (49.2)	10.37 (8.79-12.23)	11.81 (10.05-13.88)
Born 1938-1947; aged 41-80 y	355 095	179 379 (50.5)	5.39 (4.87-5.96)	7.85 (7.16-8.61)	175 716 (49.5)	5.83 (5.24-6.49)	7.95 (7.20-8.78)
Born 1928-1937; aged 51-90 y	248 970	124 614 (50.1)	2.73 (2.48-3.00)	4.92 (4.55-5.32)	124 056 (49.8)	3.03 (2.80-3.29)	5.02 (4.68-5.38)

^a^ Estimates show the associations between individuals’ index mental disorder (their first diagnosed mental disorder during the observation period) and subsequent dementia, controlling for physical diseases diagnosed before the index mental disorder. Mental disorder cases and controls were matched on observation time. Counts were randomly rounded to a base of 3 per the confidentiality rules of Statistics New Zealand. Therefore, percentages for men and women do not always sum to exactly 100% within each age band. Age ranges indicate ages during the 30-year observation period. Analyses excluded individuals who had dementia prior to their mental health diagnosis or prior to the start of their matched observation period. Hazard ratios for the associations between mental disorders and dementia across varying time intervals are shown in eAppendix 8 in the Supplement.

### Associations Across Mental Disorder Types

**Figure 3.**
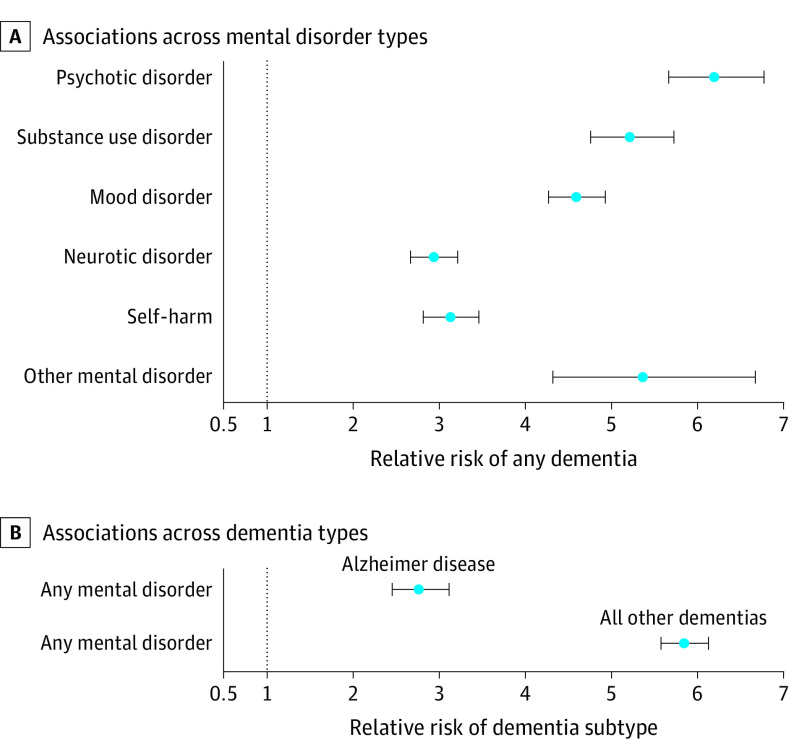
Specificity of Associations A, Mental disorders of many types were associated with subsequent onset of any dementia. The other mental disorder category includes physiological disturbance, personality, developmental, behavioral, and unspecified disorders. B, Mental disorders were associated with both Alzheimer disease and all other dementias. Dementia subtypes were ascertained using *International Statistical Classification of Diseases and Related Health Problems, Tenth Revision *(*ICD*-*10*) and corresponding *ICD*-*9* codes from public hospital and mortality records; pharmaceutical prescriptions were excluded from this ascertainment scheme because they did not specify dementia subtype. Mental disorder cases and controls were matched on observation time. Analyses excluded individuals who had dementia prior to their mental health diagnosis or prior to the start of their matched observation period. Estimates were adjusted for sex, birth year, and preexisting chronic physical disease diagnoses. Error bars indicate 95% CIs. Associations by age and sex are shown in eAppendix 10 in the Supplement.

Individuals diagnosed with psychotic, substance use, mood, neurotic, and all other mental disorders and who engaged in self-harm were all more likely than those without a mental disorder to be diagnosed with subsequent dementia, even after accounting for their physical disease histories (Figure 3A; eAppendix 10 in the Supplement). RRs ranged from 2.93 (95% CI, 2.66-3.21) for neurotic disorders to 6.20 (95% CI, 5.67-6.78) for psychotic disorders (Figure 3A).

### Associations Across Dementia Types

Associations were evident for Alzheimer disease (RR, 2.76; 95% CI, 2.45-3.11) and all other dementias (RR, 5.85; 95% CI, 5.58-6.13; Figure 3B; eAppendix 10 in the Supplement). Associations were larger for dementias other than Alzheimer, which may reflect that individuals with mental disorders are more likely to develop dementias stemming from poor health behaviors and chronic physical diseases.^30^ This difference in estimates was smallest in the earliest born cohort (eAppendix 10 in the Supplement).

### Associations Across Specifications

Pharmaceutical and mortality data were available for only portions of the observation period. To determine whether findings depended on these information sources, we reestimated associations after removing pharmaceutical and mortality records, separately, from the dementia ascertainment scheme. To determine the effect of weighting for time overseas and death, we reestimated associations using unweighted data. Associations were similar or slightly larger across specifications (eAppendices 11, 12, and 13 in the Supplement). To test whether associations were attributable to social class, we controlled for neighborhood deprivation. Associations were attenuated but still substantial (eAppendix 14 in the Supplement).

To assess the potential for unmeasured confounding to explain the association between mental disorder and dementia, we computed an E-value, which estimates what the risk ratio would need to be for an unmeasured confounder (or set of confounders) to explain away the observed association.^32^ The association between mental disorder and dementia (RR, 4.24; 95% CI, 4.07-4.42) could only be explained away by an unmeasured confounder associated with both mental disorder and dementia by a risk ratio of at least 7.95-fold each, above and beyond the measured confounders. The 95% CI could be moved to include the null only by an unmeasured confounder associated with both mental disorder and dementia by a risk ratio of at least 7.60-fold each, above and beyond the measured confounders.

## Discussion

In this nationwide health register analysis of 1.7 million citizens observed across 3 decades, individuals with mental disorders were at elevated risk of subsequent dementia and younger dementia onset. Increased risk was stronger for mental disorders than physical diseases and on par with established risks, including the *APOE4* allele.^33^ Associations were present across different mental disorders, earlier-onset and later-onset dementias, Alzheimer disease and other dementias, men and women, and all age groups and remained after accounting for preexisting physical diseases and socioeconomic deprivation.

These results have several implications. First, if associations are causal, ameliorating mental disorders in early life might mitigate neurodegenerative disease in later life. Associations were observed across different mental disorders, suggesting that preventing any disorder in early life might benefit later-life cognitive health. However, different mechanisms may connect different disorders with dementia. Depression may prompt neuroinflammation,^34^ excessive alcohol use can lead to brain damage,^35^ and psychosis may precipitate accelerated cognitive and functional decline.^36^ Characterizing shared and unique pathways of risk for dementia across different psychiatric disorders should be a research priority.

Second, mental disorders are a particularly salient antecedent of dementia among younger individuals. We observed stronger associations among the most recently born cohorts. This may reflect that individuals with mental disorders tend to die earlier than individuals without mental disorders^30^ and do not contribute dementia cases to the oldest age groups. It may also be that older individuals have more opportunity to accumulate risk factors for dementia beyond mental disorders. Our analysis cannot resolve the explanation but does reveal that poor mental health is associated with poor cognitive health among younger individuals, not just older individuals.

Third, mental disorders may be indicators of risk rather than causes of dementia. We established the temporal ordering of mental disorders before dementia, addressed reverse causation, and ruled out poor physical health and socioeconomic deprivation as alternative explanations for associations. However, there may be shared risk factors we did not measure that underlie the association between mental disorders and dementia, including a general liability to poor brain health (although twin-difference and sibling-difference analyses indicate that the association between depression and dementia holds after controlling for shared familial risks for at least these 2 conditions).^12,37,38^ Even if mental disorders do not cause dementia, they are a very early warning sign of subsequent cognitive decline, with intervention implications. Dementia is not typically treated until later life, but our findings support embedding dementia prevention into mental disorder treatment, across the life course. For instance, mental health professionals could deliver psychoeducation to clients about health behaviors to reduce dementia risk^19^ and implement interventions targeting other modifiable dementia risk factors that are more common in patients with mental disorders, such as social disconnection.^17^ Behavioral approaches are likely to be more cost-effective and consumer friendly than preventive dementia therapies, which are anticipated to be expensive, scarce, and in high demand.^39,40,41^

Fourth, our results highlight opportunities and challenges for future research on the association between psychiatric and neurological health. Opportunities include interrogating the mechanisms linking mental disorders with dementia, which could provide insights into prevention targets. If mental disorders are causal, this suggests the hypothesis that participants in psychiatric randomized clinical trials should be protected against dementia, which could be tested at long-term follow-up. A challenge will be how to retain participants with poor mental and cognitive health for follow-up into late life.

Several forms of bias and phenotype misclassification may exist. First, dementia may not be accurately diagnosed in medical records. However, our medical register–based dementia diagnoses corresponded highly with diagnoses from community-based assessments. Second, individuals with a mental disorder might have a greater opportunity to be diagnosed with dementia because they present more often for treatment or because clinicians are more likely to perform mental status examinations, including assessments of cognition for individuals with a mental disorder. Alternatively, disorders such as depression and anxiety may be more likely to be diagnosed among individuals with dementia owing to confusion regarding prodromal dementia symptoms or because physicians and families wish to target treatable mental health conditions before confirming a dementia diagnosis. These factors could have influenced our results if mental disorder and dementia diagnoses were assigned concurrently or in close succession. However, we observed associations across 30 years, with increased risk maintained over time; associations were evident for a range of mental disorders, not just depression and anxiety; and the mean time to dementia diagnosis following individuals’ first mental disorder diagnosis was 8.6 years, suggesting that in most cases, diagnoses were not assigned concurrently or in close succession. Third, the larger associations among more recently born cohorts may reflect that dementia cases are more likely to be missed among younger individuals without mental disorders because they have had less opportunity to present for treatment. However, there were only a modest number of dementia cases in the youngest cohort, and we likely captured most cases, as the cohort-level prevalence of dementia (0.1%) aligns with estimates of the global prevalence of early-onset dementia.^42^

### Limitations

This research has limitations. First, results cannot necessarily be generalized to other nations or health care systems. However, associations between some mental disorders (eg, anxiety and affective disorders) and dementia have been observed across different countries, including the US.^9,43^ We expand on prior reports by evaluating the full range of psychiatric conditions and tracking a study population with a wide age span across 30 years. Second, inpatient-hospital records will not capture less severe mental disorder cases treated on an outpatient basis. This is also the case for dementia; however, including pharmaceutical prescriptions in our ascertainment scheme likely helped to capture less severe cases. Further, associations between mental disorders and dementia have been identified using outpatient-treatment records.^12,44^ Third, our analysis, which relies on nationwide health records, does not include individuals with a mental disorder who do not receive treatment.^45,46^ Fourth, we tracked associations between mental disorders and dementia over much of the life span. However, we were missing information about later-diagnosed dementias in the youngest individuals and earlier-diagnosed mental disorders in the oldest individuals. Fifth, we accessed public hospital records rather than private hospital records. However, only about 5% of New Zealand hospitalizations occur in private hospitals, most for elective surgical procedures.^29^ Sixth, results may vary with historical differences in diagnostic practices. However, we observed associations between mental disorders and dementia among individuals born up to 39 years apart.

## Conclusions

In this study, mental disorders were associated with the onset of dementia in the population. If associations are causal, ameliorating mental disorders might benefit not only psychiatric health among younger individuals but also cognitive and functional well-being among older individuals.

## Notes

Supplement. **eAppendix 1.** Ascertainment of mental disorders. **eAppendix 2.** Ascertainment of dementia. **eAppendix 3.** Correspondence between medical register–based dementia diagnoses and dementia diagnoses ascertained from community-based assessments. **eAppendix 4.** Ascertainment of chronic physical diseases. **eAppendix 5.** Random-matching procedure. **eAppendix 6.** Prevalence of mental disorders and dementia in the study population. **eAppendix 7.** Associations between mental disorders, chronic physical diseases, and dementia by age and sex. **eAppendix 8.** Hazard ratios for the association between mental disorders and dementia across varying time intervals. **eAppendix 9.** Time to dementia diagnosis among individuals with and without a mental disorder diagnosis. **eAppendix 10.** Specificity of mental disorder and dementia associations by age and sex. **eAppendix 11.** Associations between mental disorders and subsequent dementia when excluding pharmaceutical records from the dementia ascertainment scheme. **eAppendix 12.** Associations between mental disorders and subsequent dementia when excluding mortality records from the dementia ascertainment scheme. **eAppendix 13.** Associations between mental disorders and subsequent dementia estimated using unweighted data. **eAppendix 14.** Associations between mental disorders and subsequent dementia estimated controlling for neighborhood deprivation level.
eReferences.
