# Supplementary material for: Longitudinal Associations of Mental Disorders With Dementia: 30-Year Analysis of 1.7 Million New Zealand Citizens
Source: JAMA Psychiatry. 2022 Feb 16;79(4):333–40. doi: 10.1001/jamapsychiatry.2021.4377 (PMC8851362; doi:10.1001/jamapsychiatry.2021.4377)
Supplement: Supplement. — eAppendix 1. Ascertainment of mental disorders. eAppendix 2. Ascertainment of dementia. eAppendix 3. Correspondence between medical register–based dementia diagnoses and dementia diagnoses ascertained from community-based assessments. eAppendix 4. Ascertainment of chronic physical diseases. eAppendix 5. Random-matching procedure. eAppendix 6. Prevalence of mental disorders and dementia in the study population. eAppendix 7. Associations between mental disorders, chronic physical diseases, and dementia by age and sex. eAppendix 8. Hazard ratios for the association between mental disorders and dementia across varying time intervals. eAppendix 9. Time to dementia diagnosis among individuals with and without a mental disorder diagnosis. eAppendix 10. Specificity of mental disorder and dementia associations by age and sex. eAppendix 11. Associations between mental disorders and subsequent dementia when excluding pharmaceutical records from the dementia ascertainment scheme. eAppendix 12. Associations between mental disorders and subsequent dementia when excluding mortality records from the dementia ascertainment scheme. eAppendix 13. Associations between mental disorders and subsequent dementia estimated using unweighted data. eAppendix 14. Associations between mental disorders and subsequent dementia estimated controlling for neighborhood deprivation level. eReferences. [file jamapsychiatry-e214377-s001.pdf]

Supplemental Online Content

Richmond-Rakerd LS, D’Souza S, Milne BJ, Caspi A, Moffitt TE. Longitudinal associations of mental disorders with dementia: 30-year analysis of 1.7 million New Zealand citizens. *JAMA Psychiatry*. Published online February 16, 2022. doi:10.1001/jamapsychiatry.2021.4377

eAppendix 1. Ascertainment of mental disorders

eAppendix 2. Ascertainment of dementia

eAppendix 3. Correspondence between medical register–based dementia diagnoses and dementia diagnoses ascertained from community-based assessments

eAppendix 4. Ascertainment of chronic physical diseases

eAppendix 5. Random-matching procedure

eAppendix 6. Prevalence of mental disorders and dementia in the study population

eAppendix 7. Associations between mental disorders, chronic physical diseases, and dementia by age and sex

eAppendix 8. Hazard ratios for the association between mental disorders and dementia across varying time intervals

eAppendix 9. Time to dementia diagnosis among individuals with and without a mental disorder diagnosis

eAppendix 10. Specificity of mental disorder and dementia associations by age and sex

eAppendix 11. Associations between mental disorders and subsequent dementia when excluding pharmaceutical records from the dementia ascertainment scheme

eAppendix 12. Associations between mental disorders and subsequent dementia when excluding mortality records from the dementia ascertainment scheme

eAppendix 13. Associations between mental disorders and subsequent dementia estimated using unweighted data

eAppendix 14. Associations between mental disorders and subsequent dementia estimated controlling for neighborhood deprivation level

eReferences.

This supplementary material has been provided by the authors to give readers additional information about their work.

© 2022 Richmond-Rakerd LS et al. *JAMA Psychiatry*.

eAppendix 1. Ascertainment of mental disorders

Our ascertainment scheme for mental disorders has been previously published.[1] Mental disorders were ascertained using records of diagnoses made in public hospitals, based on the International Classification of Diseases, 9th Revision (ICD-9, for admissions between July 1988 – June 1999) and 10th Revision (ICD-10, for admissions between July 1999 – June 2018). We obtained information about nine broad categories of mental disorders using the primary diagnoses for each hospital admission, based on ICD-10 and corresponding ICD-9 codes. In order to achieve consistency across diagnostic schemes, corresponding diagnoses in the ICD-9 were ascertained using mapped codes provided by the New Zealand Ministry of Health. Mapping code is hosted at the following website: https://moffittcaspi.trinity.duke.edu/research-topics/statistical-code

In the mapping code provided by the NZ Ministry of Health, ICD-9 codes 305.90, 300.4, 300.19, 300.9, 306.9, 302.89, and 307.59 mapped to ICD-10 codes for more than one diagnostic category. These codes comprised 3.9% of all ICD-9 codes in our mental-disorder ascertainment scheme. They were very low-base-rate diagnoses, and sensitivity analyses indicated that results were not dependent on the category to which they were assigned. Therefore, to maintain consistency with the mapping file, these codes were included in more than one mental-disorder category.

The physiological-disturbance, personality, developmental, behavioral, and unspecified disorders could not be analyzed as separate categories due to limited sample size and were therefore grouped in one category ("other mental disorders") for analyses.

**Mental disorders**	**Abbreviated names**	**ICD-10[a]**	**ICD-9[a]**
Mental and behavioral disorders due to psychoactive substance use	Substance use disorders	F10 – F19	291.0, 291.1, 291.8, 291.9, 292.0, 292.83, 292.89, 292.9, 303.90, 304.00, 304.10, 304.20, 304.30, 304.40, 304.50, 304.60, 304.80, 305.00, 305.1, 305.20, 305.30, 305.40, 305.50, 305.60, 305.70, 305.90
Schizophrenia and related disorders	Psychotic disorders	F20 – F29	295.00, 295.10, 295.20, 295.30, 295.40, 295.50, 295.60, 295.70, 295.80, 295.90, 297.1, 297.3, 297.8, 297.9, 298.3, 298.8, 298.9, 300.4
Mood disorders	Mood disorders	F30 – F39	296.00, 296.21, 296.22, 296.23, 296.24, 296.30, 296.31, 296.32, 296.33, 296.34, 296.36, 296.40, 296.51, 296.53, 296.54, 296.60, 296.7, 296.82, 296.90, 296.99, 300.4, 301.13, 311
Neurotic, stress-related, and somatoform disorders	Neurotic disorders	F40 – F48	300.00, 300.01, 300.02, 300.09, 300.11, 300.12, 300.13, 300.14, 300.15, 300.16, 300.19, 300.20, 300.21, 300.22, 300.23, 300.29, 300.3, 300.4, 300.5, 300.6, 300.7, 300.81, 300.89, 300.9, 306.1, 306.2, 306.4, 306.50, 306.8, 306.9, 307.80, 307.89, 308.3, 308.9, 309.29
Syndromes associated with physiological disturbances and physical factors	Physiological-disturbance disorders	F50 – F59	302.70, 302.72, 302.79, 302.89, 305.80, 305.90, 306.9, 307.1, 307.40, 307.41, 307.44, 307.45, 307.46, 307.47, 307.50, 307.51, 307.54, 307.59, 316, 648.44

© 2022 Richmond-Rakerd LS et al. *JAMA Psychiatry*.

Disorders of adult personality and behavior	Personality disorders	F60 – F69	300.19, 301.0, 301.20, 301.3, 301.4, 301.50, 301.51, 301.6, 301.7, 301.82, 301.83, 301.89, 301.9, 302.2, 302.3, 302.4, 302.50, 302.6, 302.81, 302.83, 302.89, 302.9, 312.30, 312.31, 312.33, 312.39
Pervasive and specific developmental disorders	Developmental disorders	F80 – F89	299.00, 299.10, 299.80, 299.90, 315.00, 315.1, 315.2, 315.31, 315.39, 315.4, 315.8, 315.9, 330.8
Behavioral and emotional disorders with onset usually occurring in childhood and adolescence	Behavioral disorders	F90 – F98	307.0, 307.20, 307.21, 307.22, 307.23, 307.3, 307.52, 307.59, 307.6, 307.7, 307.9, 309.21, 309.83, 312.00, 312.20, 312.89, 312.9, 313.0, 313.22, 313.89, 313.9, 314.00, 314.2, 314.8, 314.9
Unspecified mental disorders	Unspecified mental disorders	F99	300.9

[a]Diagnostic codes for substance-induced dementias were excluded from our ascertainment scheme for substance use disorders, to avoid overlap with our dementia outcome measure.

We also obtained information about self-harm using external cause codes for events of intentional self-harm (ICD-9: E950 – E959, ICD-10: X60 – X84). We excluded events of undetermined intent. Self-harm was always recorded, regardless of whether the hospital admission included a primary diagnosis of another mental disorder. If the admission included a primary diagnosis of another mental disorder, individuals were categorized as meeting criteria for both self-harm and the accompanying mental disorder.

© 2022 Richmond-Rakerd LS et al. *JAMA Psychiatry*.

eAppendix 2. Ascertainment of dementia

Alzheimer's disease and related dementias (ADRD) were ascertained using a previously-published scheme.[2] We collected information about dementias using (a) International Classification of Diseases, 10th Revision (ICD-10) and corresponding ICD-9 dementia codes in public-hospital records, (b) ICD-10 and corresponding ICD-9 dementia codes in mortality records, and (c) anti-dementia drug prescriptions in pharmaceutical records. In order to obtain consistency across diagnostic schemes, corresponding diagnoses in the ICD-9 were ascertained using mapped codes provided by the New Zealand Ministry of Health. Mapping code is hosted at the following website: https://moffittcaspi.trinity.duke.edu/research-topics/statistical-code

ICD-10 and corresponding ICD-9 codes used to ascertain dementia from public-hospital and mortality records.

**ICD-10**	**ICD-9**
F00, F00.0, F00.1, F00.2, F00.9, F01, F01.0, F01.1, F01.2, F01.3, F01.8, F01.9, F02, F02.0, F02.1, F02.2, F02.3, F02.4, F02.8, F03, F05.1, F10.7, F13.7, F18.7, F19.7, G30, G30.0, G30.1, G30.8, G30.9, G31.0, G31.1, G31.3	290.0, 290.3, 290.8, 290.9, 290.10, 290.11, 290.12, 290.13, 290.20, 290.21, 290.40, 290.41, 290.42, 290.43, 291.2, 292.82, 294.1, 331.0, 331.1, 331.2

Anti-dementia drugs (and corresponding formulation IDs) used to ascertain dementia from pharmaceutical records.
Donepezil (392325, 392326)
Rivastigmine (403725, 403726)

Note. The drugs Galantamine and Memantine are also approved for use in New Zealand to treat dementia symptoms. However, we considered only the drugs listed above as these were the drugs included in the published dementia ascertainment scheme (developed from NZ Integrated Data Infrastructure health records) that we followed for the current report.[2]

© 2022 Richmond-Rakerd LS et al. *JAMA Psychiatry*.

eAppendix 3. Correspondence between medical register–based dementia diagnoses and dementia diagnoses ascertained from community-based assessments

Records from public hospitals, death certificates, and pharmaceutical dispensing will fail to capture many dementia cases that are less severe and are treated on an outpatient basis. We therefore assessed the correspondence between our medical register-based dementia diagnoses with diagnoses collected from the international Residential Assessment Instrument (interRAI) database, maintained by the New Zealand Ministry of Health. The InterRAI is a geriatric assessment instrument developed by health researchers in over 30 countries.[3] Since 2012, the interRAI has been administered to individuals in New Zealand requiring publicly-funded, long-term community services or aged residential care.[3] We restricted analyses to records starting in 2016 because use of the interRAI for aged residential care assessments was not implemented across all district health boards until 2016 (Hamish Jamieson, PhD, personal communication, July 2021).

A total of 90,792 individuals in our study population were also identified in the interRAI database. Shown below are the numbers of individuals diagnosed with dementia via our medical register-based ascertainment scheme and via the interRAI assessment, as well as the corresponding positive and negative predictive values.

	**InterRAI**	
**Medical registers**	No dementia	Dementia
No dementia	64,905	10,578
Dementia	2,589	12,726

Positive predictive value = 83.1%
Negative predictive value = 86.0%

Notes. Counts were randomly rounded to a base of three per the confidentiality rules of Statistics New Zealand.

© 2022 Richmond-Rakerd LS et al. *JAMA Psychiatry*.

eAppendix 4. Ascertainment of chronic physical diseases

Our ascertainment scheme for chronic physical diseases has been previously published.[1] We obtained information about eight chronic and age-related physical diseases, which comprise the diseases included in the Chronic Conditions Data Dictionary maintained by the New Zealand Ministry of Health (available at http://archive.stats.govt.nz/browse_for_stats/snapshots-of-nz/integrated-data-infrastructure/idi-data/chron-condn-sig-health-evt-data.aspx#gsc.tab=0). The dictionary documents procedures for ascertaining chronic conditions from New Zealand's health registers. We coded each disease using the primary diagnoses for each hospital admission (and procedure codes for coronary heart disease), based on the ICD-9 and ICD-10 codes provided in the data dictionary. The codes are listed below. Where ICD-9 codes were not provided, we used mapped codes provided by the New Zealand Ministry of Health. Mapping code is hosted at the following website: https://moffittcaspi.trinity.duke.edu/research-topics/statistical-code.

Per the procedures outlined in the data dictionary, both coronary heart disease and myocardial infarction were ascertained using ICD-10 code I21 and ICD-9 code 410. If these codes were given as the primary diagnosis, individuals were diagnosed with both conditions. Because coronary heart disease was also ascertained via procedure codes, occasionally a different physical disease was coded as the primary diagnosis for the same hospital admission. In these cases, individuals were diagnosed with both conditions.

**Physical diseases**	**ICD-10**	**ICD-9**
Coronary heart disease[a]	Primary diagnoses:<br>I20 – I25, Z95.1, Z95.5<br><br>Procedure codes:<br>3530400, 3530500, 3531000, 3531001, 3531002, 3849700, 3849701, 3849702, 3849703, 3849704, 3849705, 3849706, 3849707, 3850000, 3850001, 3850002, 3850003, 3850004, 3850300, 3850301, 3850302, 3850303, 3850304, 3863700, 9020100, 9020101, 9020102, 9020103	Primary diagnoses:<br>410 – 414, V45.81, V45.82<br><br>Procedure codes:<br>36.01, 36.02, 36.03, 36.04, 36.05, 36.06, 36.07, 36.10, 36.11, 36.12, 36.13, 36.14, 36.15, 36.16
Gout	M10	274
Chronic obstructive pulmonary disease	J40, J41.0, J41.1, J41.8, J42, J43.0, J43.1, J43.2, J43.8, J43.9, J44.0, J44.1, J44.8, J44.9	490, 491.0, 491.1, 491.20, 491.21, 491.8, 491.9, 492.0, 492.8, 496
Diabetes	E10, E11, E13, E14, O24.0, O24.1, O24.2, O24.3	Mapped codes: 250.00, 250.01, 250.02, 250.03, 250.10, 250.11, 250.12, 250.13, 250.30, 250.31, 250.32, 250.33, 250.40, 250.41, 250.42, 250.43, 250.50, 250.51, 250.52, 250.53, 250.60, 250.61, 250.62, 250.63, 250.70, 250.71, 250.72, 250.73, 250.80, 250.81, 250.82, 250.83, 250.90, 250.91, 250.92, 250.93, 648.01, 648.03, 648.04
Cancer	C00 – C96, D45 – D47	Mapped codes: 140.0, 140.1, 140.3, 140.4, 140.6, 140.8, 140.9, 141.0, 141.1, 141.2, 141.3, 141.4, 141.6, 141.8, 141.9, 142.0, 142.1, 142.2, 142.8, 142.9, 143.0, 143.1, 143.9, 144.0, 144.1, 144.8, 144.9, 145.0,

© 2022 Richmond-Rakerd LS et al. *JAMA Psychiatry*.

		145.1, 145.2, 145.3, 145.4, 145.5, 145.6, 145.8, 145.9, 146.0, 146.1, 146.2, 146.3, 146.4, 146.6, 146.7, 146.8, 146.9, 147.0, 147.1, 147.2, 147.3, 147.8, 147.9, 148.0, 148.1, 148.2, 148.3, 148.8, 148.9, 149.0, 149.8, 150.0, 150.1, 150.2, 150.3, 150.4, 150.5, 150.8, 150.9, 151.0, 151.1, 151.2, 151.3, 151.4, 151.5, 151.6, 151.8, 151.9, 152.0, 152.1, 152.2, 152.3, 152.8, 152.9, 153.0, 153.1, 153.2, 153.3, 153.4, 153.5, 153.6, 153.7, 153.8, 153.9, 154.0, 154.1, 154.2, 154.3, 154.8, 155.0, 155.1, 156.0, 156.1, 156.2, 156.8, 156.9, 157.0, 157.1, 157.2, 157.3, 157.4, 157.8, 157.9, 158.0, 158.8, 158.9, 159.0, 159.1, 159.8, 159.9, 160.0, 160.1, 160.2, 160.3, 160.4, 160.5, 160.8, 160.9, 161.0, 161.1, 161.2, 161.3, 161.8, 161.9, 162.0, 162.2, 162.3, 162.4, 162.5, 162.8, 162.9, 163.9, 164.0, 164.1, 164.2, 164.3, 164.8, 164.9, 165.0, 165.8, 165.9, 170.01, 170.02, 170.1, 170.2, 170.3, 170.4, 170.5, 170.6, 170.7, 170.8, 170.9, 171.0, 171.2, 171.3, 171.4, 171.5, 171.6, 171.7, 171.8, 171.9, 172.0, 172.1, 172.2, 172.3, 172.4, 172.5, 172.6, 172.7, 172.8, 172.9, 173.0, 173.1, 173.2, 173.3, 173.4, 173.5, 173.6, 173.7, 173.8, 173.9, 174.0, 174.1, 174.2, 174.3, 174.4, 174.5, 174.6, 174.8, 174.9, 176.0, 176.1, 176.2, 176.5, 176.8, 176.9, 179, 180.0, 180.1, 180.8, 180.9, 181, 182.0, 182.1, 182.8, 183.0, 183.2, 183.3, 183.4, 183.5, 183.9, 184.0, 184.1, 184.2, 184.3, 184.4, 184.8, 184.9, 185, 186.0, 186.9, 187.1, 187.2, 187.3, 187.4, 187.5, 187.6, 187.7, 187.8, 187.9, 188.0, 188.1, 188.2, 188.3, 188.4, 188.5, 188.6, 188.7, 188.8, 188.9, 189.0, 189.1, 189.2, 189.3, 189.8, 189.9, 190.0, 190.1, 190.2, 190.3, 190.4, 190.5, 190.6, 190.8, 190.9, 191.0, 191.1, 191.2, 191.3, 191.4, 191.5, 191.6, 191.7, 191.8, 191.9, 192.0, 192.1, 192.2, 192.3, 192.8, 192.9, 193, 194.0, 194.1, 194.3, 194.4, 194.5, 194.6, 194.9, 195.0, 195.1, 195.2, 195.3, 195.4, 195.5, 195.8, 196.0, 196.1, 196.2, 196.3, 196.5, 196.6, 196.8, 196.9, 197.0, 197.1, 197.2, 197.3, 197.4, 197.5, 197.6, 197.7, 197.8, 198.0, 198.1, 198.2, 198.3, 198.4, 198.5, 198.6, 198.7, 198.81, 198.82, 198.89, 199.1, 200.00, 200.10, 200.20, 200.80, 201.00, 201.40, 201.50, 201.60, 201.70, 201.90, 202.00,202.10, 202.20, 202.30, 202.40, 202.41, 202.50, 202.60, 202.80, 202.90, 203.00, 203.01, 203.10, 203.11, 203.80, 204.00, 204.01, 204.10, 204.11, 204.20, 204.80, 204.81, 204.90, 204.91, 205.00, 205.01, 205.10, 205.11, 205.20, 205.21, 205.30, 205.31, 205.80, 205.81, 205.90, 205.91, 206.00, 206.01, 206.10, 206.11, 206.80, 206.90, 206.91, 207.00, 207.01, 207.10, 207.20, 207.21, 207.80, 207.81, 208.00, 208.01, 208.10, 208.80, 208.90, 208.91, 238.4, 238.5, 238.6, 238.7, 273.1, 273.2, 273.3, 289.8
Traumatic brain injury	S06	800 – 801.9, 803 – 804.9, 850 – 854

© 2022 Richmond-Rakerd LS et al. *JAMA Psychiatry*.

Stroke	I60 – I64	430 – 432, 433.01, 433.11, 433.21, 433.31, 433.81, 433.91, 434.01, 434.11, 434.91, 436
Myocardial infarction	I21	410

[a]Following the procedures given in the New Zealand Ministry of Health's Chronic Conditions Data Dictionary, procedure codes (in addition to primary diagnoses) were used to ascertain coronary heart disease.

© 2022 Richmond-Rakerd LS et al. *JAMA Psychiatry*.

eAppendix 5. Random-matching procedure

Our random-matching procedure has been previously published.[1] We assessed whether mental-disorder hospitalizations between July 1988-June 2018 were associated with subsequent dementia diagnoses during the period. Our analysis needed to account for the different duration of observation time among cases (those with a mental disorder, who were observed from their first mental-health hospitalization, which could occur at any time during the study period) and controls (those without a mental disorder, whose observation time was the full 30 years). Failing to account for differing observation periods could lead to biased estimates, because controls would have a greater opportunity to develop a dementia diagnosis than cases.

To address this problem, we randomly assigned observation periods to controls to match the observation periods of cases, based on the distributions of admission dates for cases' mental-disorder hospitalizations. For example, suppose .53%, .37%, and .41% of cases had their first mental-disorder hospitalization in July 1992, March 1998, and October 2015, respectively. In this scenario, we would randomly assign .53% of controls to have their observation period start in July 1992, .37% to have their observation period start in March 1998, and .41% to have their observation period start in October 2015. Importantly, we only recorded dementia diagnoses during these truncated observation periods, ignoring all diagnoses prior to the randomly-assigned starting months. We took this approach for each of the 360 months from July 1988 to June 2018, and so created a cohort of controls with the same distribution of observation periods as cases.

Finally, to account for potentially different distributions of mental-disorder hospitalizations across age, we used birth cohort-specific distributions of start dates among cases to randomly assign start dates to controls. That is, the distribution of start dates among cases born in 1928-37 was used to randomly assign start dates to controls born in 1928-37, the distribution of start dates among cases born in 1938-47 was used to randomly assign start dates to controls born in 1938-47, etc.

eAppendix 6. Prevalence of mental disorders and dementia in the study population

	Total study population	
	**Mental disorder**	**Dementia**
Total (N=1,711,386)	3.8	2.0
Men (N=866,301)	3.9	2.0
Women (N=845,085)	3.7	2.0
	**Men**	
	**Mental disorder**	**Dementia**
Born 1958-67 (N=305,556)	4.8	0.1
Born 1948-57 (N=254,214)	3.7	0.5
Born 1938-47 (N=180,009)	3.0	2.5
Born 1928-37 (N=126,516)	3.0	8.6
	**Women**	
	**Mental disorder**	**Dementia**
Born 1958-67 (N=295,938)	4.6	0.1
Born 1948-57 (N=246,459)	3.4	0.4
Born 1938-47 (N=176,223)	3.0	2.2
Born 1928-37 (N=126,465)	3.3	9.4

Notes. Counts were randomly rounded to a base of three per the confidentiality rules of Statistics New Zealand. Therefore, counts do not always sum to totals.

© 2022 Richmond-Rakerd LS et al. *JAMA Psychiatry*.

eAppendix 7. Associations between mental disorders, chronic physical diseases, and dementia by age and sex

A. Baseline bivariate models[a]

Age band	Men	Women
	Any mental disorder	
Born 1958-67	8.30 [6.56, 10.50]	9.32 [7.12, 12.20]
Born 1948-57	7.65 [6.72, 8.70]	7.98 [6.86, 9.29]
Born 1938-47	4.73 [4.34, 5.16]	4.93 [4.49, 5.42]
Born 1928-37	2.51 [2.33, 2.70]	2.55 [2.39, 2.73]
	Any physical disease	
Born 1958-67	2.80 [2.21, 3.56]	2.05 [1.48, 2.84]
Born 1948-57	1.77 [1.58, 1.98]	2.35 [2.05, 2.69]
Born 1938-47	1.40 [1.32, 1.48]	1.67 [1.57, 1.78]
Born 1928-37	1.11 [1.07, 1.16]	1.11 [1.07, 1.15]

B. Multivariate models[b]

Age band	Men	Women
	Any mental disorder	
Born 1958-67	7.23 [5.69, 9.20]	8.80 [6.69, 11.59]
Born 1948-57	7.06 [6.19, 8.06]	6.88 [5.89, 8.04]
Born 1938-47	4.50 [4.13, 4.92]	4.44 [4.03, 4.89]
Born 1928-37	2.48 [2.30, 2.67]	2.52 [2.36, 2.70]
	Any physical disease	
Born 1958-67	2.13 [1.67, 2.72]	1.45 [1.04, 2.02]
Born 1948-57	1.47 [1.30, 1.65]	1.87 [1.62, 2.14]
Born 1938-47	1.30 [1.23, 1.38]	1.50 [1.40, 1.60]
Born 1928-37	1.09 [1.05, 1.13]	1.07 [1.04, 1.11]

Notes. Estimates are relative risks. 95% confidence limits are in brackets. We ascertained mental-disorder and physical-disease diagnoses during the 30-year observation period. To be included in analyses, dementia diagnoses had to occur subsequent to the mental-disorder or physical-disease diagnosis.
[a]Mental disorders and physical diseases were entered as predictors in separate models.
[b]Mental disorders and physical diseases were entered together as predictors in the same model.

© 2022 Richmond-Rakerd LS et al. *JAMA Psychiatry*.

eAppendix 8. Hazard ratios for the association between mental disorders and dementia across varying time intervals

We calculated hazard ratios for the association between mental disorders and dementia across different follow-up intervals, in the total study population. As shown below, increased risk for dementia was maintained across all lengths of follow-up.

Time interval (length of observation)					
**1 year**	**5 years**	**10 years**	**15 years**	**20 years**	**25 years**
6.81 [6.13, 7.56]	5.54 [5.22, 5.88]	5.22 [4.98, 5.48]	5.19 [4.97, 5.42]	5.20 [5.00, 5.42]	5.37 [5.17, 5.58]

Notes. Estimates are hazard ratios [and 95% confidence intervals]. Models controlled for sex, birth year, and pre-existing chronic physical diseases. Mental-disorder cases and controls were matched on observation time. Analyses excluded individuals who had dementia prior to their mental-health diagnosis or prior to the start of their matched observation period. For all time intervals, follow-up started at the index mental-disorder diagnosis (for cases) or the start of the matched observation period (for controls).

© 2022 Richmond-Rakerd LS et al. *JAMA Psychiatry*.

eAppendix 9. Time to dementia diagnosis among individuals with and without a mental disorder diagnosis

We compared the mean time-to-dementia diagnosis (time-to-event) among individuals with versus without a mental-disorder diagnosis during the 30-year observation period. Among individuals who developed dementia during the observation period, those with a mental disorder developed dementia 5.60 [5.31, 5.90] years earlier, on average, than those without a mental disorder (mean time-to-event: mental disorder = 8.56 years, no mental disorder = 14.17 years). (Note: The mean-difference estimate is 5.60 instead of 5.61 due to rounding.) Estimates are adjusted for pre-existing chronic physical diseases, sex, and birth year. The unadjusted estimates were very similar (mental disorder = 8.89 years, no mental disorder = 14.13 years, difference = 5.24 [4.95, 5.53] years).

© 2022 Richmond-Rakerd LS et al. *JAMA Psychiatry*.

eAppendix 10. Specificity of mental disorder and dementia associations by age and sex

A. By mental-disorder type

	Psychotic disorder	Substance use disorder	Mood disorder	Neurotic disorder	Self-harm	Other mental disorder
Men	6.41 [5.61, 7.32]	5.24 [4.70, 5.85]	4.64 [4.17, 5.18]	2.84 [2.43, 3.32]	2.72 [2.34, 3.16]	6.29 [4.73, 8.37]
Women	5.99 [5.30, 6.76]	5.06 [4.26, 6.00]	4.53 [4.12, 4.98]	2.95 [2.63, 3.32]	3.61 [3.12, 4.19]	4.42 [3.15, 6.19]
Born 1958-67	18.65 [13.29, 26.18]	14.29 [9.99, 20.44]	12.46 [8.59, 18.06]	5.73 [3.14, 10.44]	5.71 [3.87, 8.44]	13.53 [7.10, 25.82]
Born 1948-57	13.52 [10.99, 16.64]	12.47 [10.09, 15.40]	11.92 [9.88, 14.37]	6.92 [5.27, 9.08]	5.64 [4.47, 7.13]	11.99 [7.66, 18.79]
Born 1938-47	8.06 [6.91, 9.41]	6.52 [5.56, 7.66]	6.64 [5.86, 7.51]	4.13 [3.48, 4.89]	3.07 [2.52, 3.74]	7.49 [5.16, 10.86]
Born 1928-37	3.88 [3.36, 4.48]	3.29 [2.84, 3.81]	3.14 [2.83, 3.48]	2.29 [2.02, 2.60]	2.42 [2.06, 2.84]	2.94 [1.97, 4.37]

B. By dementia type

	Alzheimer's disease	All other dementias
Men	2.63 [2.19, 3.16]	6.00 [5.62, 6.40]
Women	2.86 [2.44, 3.34]	5.64 [5.28, 6.03]
Born 1958-67	2.57 [1.01, 6.53]	16.97 [13.41, 21.49]
Born 1948-57	5.63 [4.10, 7.73]	16.84 [14.79, 19.19]
Born 1938-47	2.98 [2.37, 3.76]	9.12 [8.40, 9.91]
Born 1928-37	2.37 [2.03, 2.78]	3.63 [3.39, 3.89]

Notes. Estimates are relative risks. 95% confidence limits are in brackets. Models controlled for chronic physical diseases diagnosed before the index mental disorder. Models estimated within sex controlled for birth year; models estimated within birth year controlled for sex. Mental-disorder cases and controls were matched on observation time. Analyses excluded individuals who had dementia prior to their mental-health diagnosis or prior to the start of their matched observation period. A, "other mental disorder" category includes physiological-disturbance, personality, developmental, behavioral, and unspecified disorders. B, dementia subtypes were ascertained using ICD-10 and corresponding ICD-9 codes from public-hospital and mortality records; pharmaceutical prescriptions were excluded from this ascertainment scheme.

© 2022 Richmond-Rakerd LS et al. *JAMA Psychiatry*.

eAppendix 11. Associations between mental disorders and subsequent dementia when excluding pharmaceutical records from the dementia ascertainment scheme

	**Total study population**	
	4.94 [4.73, 5.15]	
	**Men**	**Women**
Born 1958-67	13.16 [9.77, 17.72]	15.08 [10.81, 21.04]
Born 1948-57	13.51 [11.57, 15.77]	13.71 [11.39, 16.49]
Born 1938-47	7.12 [6.41, 7.91]	7.63 [6.81, 8.55]
Born 1928-37	3.04 [2.76, 3.35]	3.30 [3.04, 3.59]

Notes. Estimates are relative risks [and 95% confidence intervals]. Models controlled for chronic physical diseases diagnosed before the index mental disorder. Models estimated within the total study population also controlled for sex and birth year. Mental-disorder cases and controls were matched on observation time. Analyses excluded individuals who had dementia prior to their mental-health diagnosis or prior to the start of their matched observation period.

© 2022 Richmond-Rakerd LS et al. *JAMA Psychiatry*.

eAppendix 12. Associations between mental disorders and subsequent dementia when excluding mortality records from the dementia ascertainment scheme

	**Total study population**	
	4.25 [4.08, 4.43]	
	**Men**	**Women**
Born 1958-67	10.21 [7.81, 13.35]	10.84 [7.99, 14.71]
Born 1948-57	9.32 [8.07, 10.76]	10.45 [8.85, 12.34]
Born 1938-47	5.37 [4.85, 5.94]	5.88 [5.28, 6.54]
Born 1928-37	2.72 [2.48, 3.00]	3.02 [2.78, 3.28]

Notes. Estimates are relative risks [and 95% confidence intervals]. Models controlled for chronic physical diseases diagnosed before the index mental disorder. Models estimated within the total study population also controlled for sex and birth year. Mental-disorder cases and controls were matched on observation time. Analyses excluded individuals who had dementia prior to their mental-health diagnosis or prior to the start of their matched observation period.

© 2022 Richmond-Rakerd LS et al. *JAMA Psychiatry*.

eAppendix 13. Associations between mental disorders and subsequent dementia estimated using unweighted data

	Total study population	
	4.16 [4.02, 4.32]	
	**Men**	**Women**
Born 1958-67	10.29 [7.99, 13.24]	11.30 [8.46, 15.08]
Born 1948-57	9.34 [8.16, 10.69]	9.89 [8.44, 11.58]
Born 1938-47	5.70 [5.21, 6.24]	5.86 [5.32, 6.46]
Born 1928-37	2.99 [2.77, 3.22]	3.06 [2.86, 3.27]

Notes. Estimates are relative risks [and 95% confidence intervals]. Models controlled for chronic physical diseases diagnosed before the index mental disorder. Models estimated within the total study population also controlled for sex and birth year. Mental-disorder cases and controls were matched on observation time. Analyses excluded individuals who had dementia prior to their mental-health diagnosis or prior to the start of their matched observation period.

© 2022 Richmond-Rakerd LS et al. *JAMA Psychiatry*.

eAppendix 14. Associations between mental disorders and subsequent dementia estimated controlling for neighborhood deprivation level

	**Total study population**	
	2.85 [2.73, 2.98]	
	**Men**	**Women**
Born 1958-67	7.13 [5.29, 9.61]	7.88 [5.68, 10.93]
Born 1948-57	7.12 [6.08, 8.33]	6.88 [5.76, 8.23]
Born 1938-47	4.26 [3.82, 4.74]	3.82 [3.41, 4.28]
Born 1928-37	1.94 [1.76, 2.14]	1.89 [1.74, 2.06]

Notes. Neighborhood deprivation was assessed using the 2013 New Zealand Deprivation Index (NZDep2013)[4], an area-level measure of deprivation. The NZDep2013 assigns census areas a deprivation decile value ranging from 1 (least deprived) to 10 (most deprived) based on socioeconomic indicators from the 2013 New Zealand Census. Neighborhood deprivation information was available from 1999 to the end of the study period (2018). For individuals who changed addresses, we averaged across the deprivation levels for up to the first 20 addresses.

Models controlled for chronic physical diseases diagnosed before the index mental disorder. Models estimated within the total study population also controlled for sex and birth year. Mental-disorder cases and controls were matched on observation time. Analyses excluded individuals who had dementia prior to their mental-health diagnosis or prior to the start of their matched observation period.

© 2022 Richmond-Rakerd LS et al. *JAMA Psychiatry*.

eReferences.

[1]Richmond-Rakerd LS, D'Souza S, Milne BJ, Caspi A, Moffitt TE. Longitudinal associations of mental disorders with physical diseases and mortality in 2.3 million New Zealand citizens. JAMA Netw Open. 2021;4(1):e2033448.

[2]Walesby KE, Exeter DJ, Gibb S, et al. Prevalence and geographical variation of dementia in New Zealand from 2012 to 2015: Brief report utilising routinely collected data within the Integrated Data Infrastructure. Australas J Ageing. 2020;39(3):297-304.

[3]Schluter PJ, Ahuriri-Driscoll A, Anderson TJ, et al. Comprehensive clinical assessment of home-based older persons within New Zealand: An epidemiological profile of a national cross-section. Aust N Z J Public Health. 2016;40(4):349-355.

[4]Atkinson J, Salmond C, Crampton P. NZDep2013 index of deprivation. University of Otago, Wellington. May 2014:1-64.

© 2022 Richmond-Rakerd LS et al. *JAMA Psychiatry*.
